# Primary Malignant Peripheral Nerve Sheath Tumor of the Cauda Equina: A Case Report and Literature Review

**DOI:** 10.7759/cureus.77096

**Published:** 2025-01-07

**Authors:** Hoai TP. Dinh, Vu H Nguyen, Dung TM. Nguyen, Minh T Nguyen, Yoko Kato

**Affiliations:** 1 Department of Neurosurgery, Hue University of Medicine and Pharmacy, Hue University, Hue, VNM; 2 Department of Orthopaedic Surgery, Hamamatsu University School of Medicine, Hamamatsu, JPN; 3 Department of Neurosurgery, Fujita Health University, Toyoake, JPN

**Keywords:** cauda equina, gross total resection, malignant tumor, peripheral nerve sheath tumor, radiotherapy

## Abstract

Primary malignant peripheral nerve sheath tumors (MPNSTs) arising within the cauda equina are exceptionally rare, with only 24 cases documented in English-language literature. Due to its infrequency and aggressive behavior, no standardized treatment approach has been established. This report presents a case of primary MPNST of the cauda equina, accompanied by a comprehensive literature review, aiming to elucidate the management strategies and prognosis of this uncommon yet highly malignant tumor.

A 62-year-old male was diagnosed with primary intradural MPNST and underwent gross total resection (GTR) with laminectomy along with adjunctive high-energy radiotherapy. Concurrently, we analyze existing literature concerning intradural MPNSTs.

Surgical resection remains the mainstay of MPNST management, although its efficacy is limited by high recurrence rates. Despite aggressive treatment modalities, including radiotherapy and chemotherapy, primary intradural MPNSTs exhibit a propensity for leptomeningeal and systemic dissemination, contributing to a dismal overall prognosis. Notably, outcomes appear to be graver compared to MPNSTs in other anatomical locations.

Primary intradural MPNSTs represent a rare and formidable clinical challenge characterized by poor prognostic outcomes. While surgical excision supplemented by adjuvant radiotherapy may offer some benefit, the need for effective targeted therapies associated with neurofibromatosis type 1 (NF1) needs to be studied more to delineate optimal treatment strategies and improve patient outcomes.

## Introduction

Malignant peripheral nerve sheath tumors (MPNSTs) are rare, aggressive soft tissue sarcomas that exhibit differentiation toward nerve sheath elements. Comprising approximately 5%-10% of all soft tissue sarcomas, these tumors have an estimated lifetime incidence of about 0.001% (one in 100,000) in the general population [1]. Malignant peripheral nerve sheath tumors typically originate in peripheral nerves and are most commonly located in the trunk and extremities, associated with neurofibromatosis type 1 (NF1) in roughly 50% of cases​ [2].

Primary intradural MPNSTs, particularly those affecting the cauda equina, are exceedingly rare, with only 24 cases reported in the English literature [3]. These tumors are challenging to diagnose and treat, given their resemblance to more benign intradural spinal tumors, such as schwannomas or neurofibromas, on imaging. Complete surgical resection remains the mainstay of treatment; however, the tendency of MPNSTs to recur and metastasize often limits long-term success. This recurrence is partly attributed to their aggressive nature and the high potential for leptomeningeal spread.

The etiology of MPNSTs involves complex genetic mutations, including those affecting NF1, CDKN2A, and polycomb repressive complex 2 (PRC2) genes, such as EED and SUZ12 [4]. Understanding these mutations has opened the door to potential molecular-targeted therapies, though the application of these treatments is still under investigation. Furthermore, recent advances in radiotherapy, such as carbon ion radiotherapy (CIRT), have shown promise in controlling tumor growth for MPNSTs with resistant features, although their use in primary intradural MPNSTs remains limited to case reports​ [5].

This case report details a rare instance of a primary intradural MPNST in the cauda equina of a 62-year-old male. We present the clinical presentation, diagnostic workup, surgical approach, postoperative outcomes, and adjuvant therapy. A literature review follows to contextualize this case within the broader spectrum of MPNST management, emphasizing the challenges and future research directions for this rare and aggressive tumor.

## Case presentation

**Figure 1:**
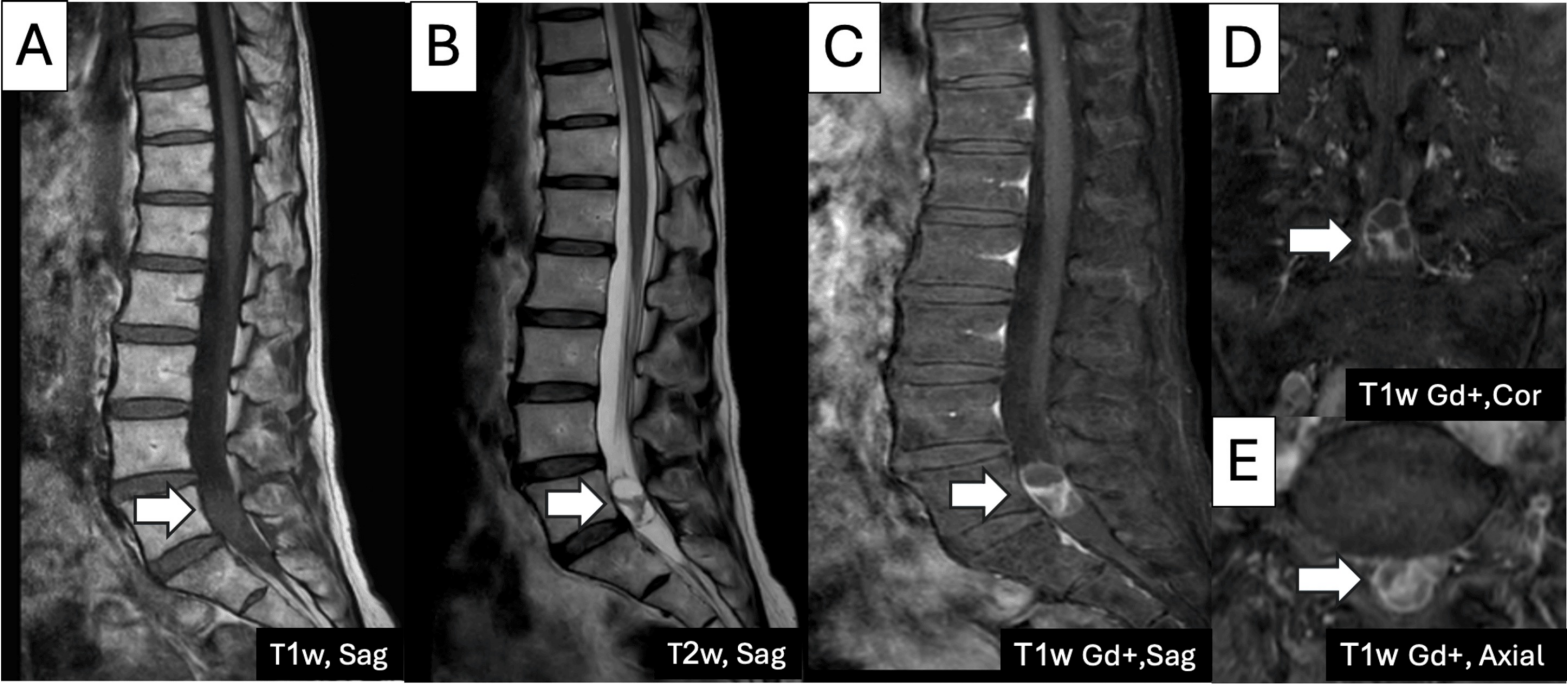
The MRI of the lumbar spine reveals a heterogeneously well-circumscribed mass at the L5 level that is iso-intense on T1-weighted (T1W) image (A) and hyper-intense on T2-weighted (T2W) image (B), enhanced after the contrast injection on the sagittal plane (C), coronal plane (D), and axial plane (E).

A 62-year-old male presented to our neurosurgery clinic with complaints of persistent gluteal and posterior femoral pain, often requiring a walking cane for ambulation. The patient also reported intermittent difficulty with urination. Neurological examination revealed normal motor power, and Babinski's reflexes were negative. Gait assessment indicated reliance on a walking aid, but no significant motor deficits were detected. Magnetic resonance imaging (MRI) of the lumbar spine revealed a well-circumscribed, heterogeneous mass at the L5 level. On T1-weighted images, the mass appeared iso-intense, whereas on T2-weighted images, it showed hyperintensity. After gadolinium contrast administration, the mass demonstrated enhancement. The lesion was intradural, extramedullary, centrally located, and slightly displaced to the left (Figure 1). Based on imaging characteristics, differential diagnoses included intradural extramedullary tumors such as schwannoma or neurofibroma.

**Figure 2:**
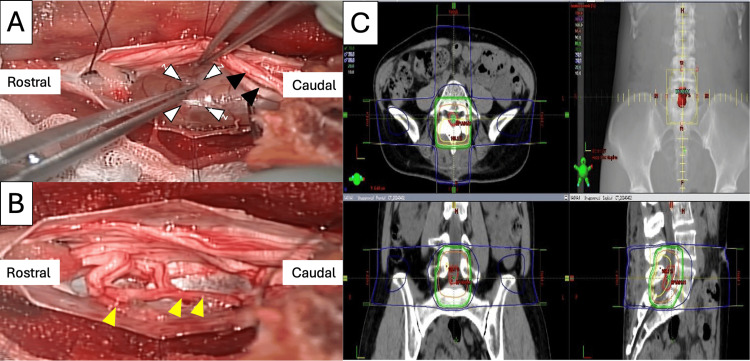
(A) Intraoperative image with a well-defined tumor (white arrowhead) compressing the L5 nerve root (black arrowhead). (B) The tumor is completely removed with the preservation of the nerve root (yellow arrowhead). (C) After determining the histopathological type of MPNST, the patient undergoes radiation therapy at the L5 level.

The patient underwent a laminectomy from L4 to S1 via a posterior approach. The dura mater was meticulously opened, revealing a soft, yellow-fluid-containing tumor at the L5 level. The mass adhered to the filum terminale and was closely associated with the dorsal rootlets of the left L5 spinal nerves. Intraoperative neurophysiological monitoring (somatosensory evoked potentials and motor evoked potentials) was used to preserve nerve function throughout the procedure. The tumor was carefully dissected from the surrounding nerve roots and was completely excised. Reconstruction involved dural repair and closure of the laminectomy site from L4 to S1 (Figure 2).

**Figure 3:**
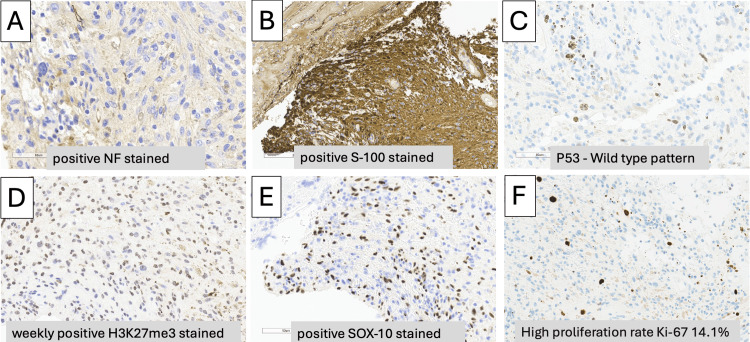
Histopathology shows positive for nuclear factor (NF) (A), S-100 (B), P53 (C), H3K27me3 (D), SOX-10 (E), and a high proliferation rate with Ki-67 14.1% (F).

Pathological examination of the excised tumor confirmed a diagnosis of MPNST. Immunohistochemical staining showed positive results for S-100 and SOX-10, consistent with nerve sheath differentiation. The tumor exhibited a high proliferation index with a Ki-67 labeling rate of 14.1%. Additional markers included nuclear factor (NF), which was positive, and p53, showing wild-type expression. H3K27me3 staining was weakly positive, further supporting the MPNST diagnosis (Figure 3).

**Figure 4:**
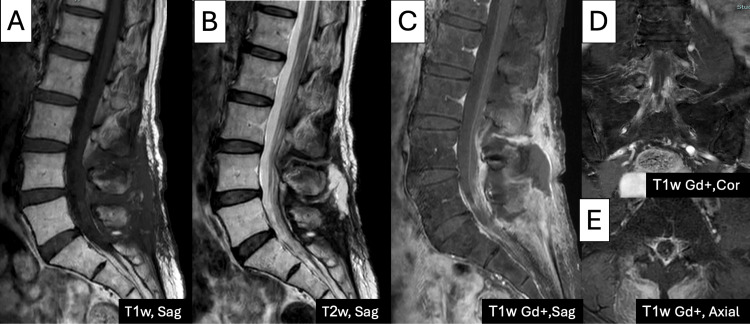
An MRI obtained nine months after surgery showed that the extradural intramedullary tumor was completely removed on the sagittal plane on T1-weighted (T1W) image (A), on T2-weighted (T2W) image (B), and after contrast injection with the sagittal plane (C), coronal plane (D), and axial plane (E).

Postoperatively, the patient had an unremarkable recovery and remained neurologically intact, with no observed complications. Follow-up MRI confirmed complete resection of the intradural extramedullary mass (Figure 4). The patient was subsequently referred for adjuvant high-dose radiotherapy, receiving a total of 50 Gy over 25 fractions. Dose constraints for adjacent organs, including the bowel, were applied as follows: the volume of bowel receiving ≥40 Gy was kept below 30% to reduce toxicity risks. During follow-up, the patient did not report significant gastrointestinal (GI) toxicity, with no episodes of diarrhea, nausea, or abdominal discomfort observed during or after treatment. No recurrence or further complications were noted at follow-up evaluations three months postoperatively.

## Discussion

Literature review

Malignant peripheral nerve sheath tumors are rare and highly aggressive soft tissue sarcomas with nerve sheath differentiation. Malignant peripheral nerve sheath tumors comprise about 5%-10% of all soft tissue sarcomas, with a lifetime incidence of approximately 0.001%, or one in 100,000 individuals​ [1]. The average age of onset is around 30-50 years, although, in NF1 patients, it may occur 10 years earlier on average. It occurs mainly in the proximal limbs, followed by the trunk, head, and neck. The main clinical manifestations are pain and numbness; however, they are not specific symptoms, and MPNSTs are difficult to distinguish from other nerve lesions [6]. Malignant peripheral nerve sheath tumors are still difficult to diagnose and treat, and the overall prognosis is poor. Primary intradural MPNSTs, such as in the cauda equina, are exceedingly rare. Only 24 cases of primary intradural MPNSTs have been reported in the English literature, and these cases represent a unique subset with specific clinical and therapeutic challenges (Table 1).

**Table 1:** Review based on the literature about malignant peripheral nerve sheath tumors (MPNSTs) m: male; F: female; GTR: gross total resection; STR: subtotal resection; NA: not applicable

No.	Reference	Age/sex	Neurofibromatosis	Vertebral	Operation	Adjuvant	Recurrence	Metastasis	Outcome
1	Thomeer et al., 1981 [7]	42/M	No	Cauda equina	GTR	Radiotherapy, Chemotherapy	Yes	No	Alive at three years with recurrence
2	Valdueza et al., 1991 [8]	43/F	No	Thoracic	STR	Radiotherapy	Yes	No	Alive at 10 years with recurrence
		47/M	Yes	Cervical	GTR	Radiotherapy	Yes	Brain	Dead at 18 months
		18/M	Na	Cervical	GTR	No	No	No	Alive and disease-free at eight months
		70/F	No	Cervical	GTR	No	No	No	Alive and disease-free at seven months
6	Seppala et al., 1993 [9]	13/M	Yes	Lumbar	STR	NA	Yes	Brain, system	Dead at two months
		13/M	No	Lumbar	GTR	NA	Yes	System	Dead at seven months
		35/M	No	Lumbar	GTR	NA	Yes	System	Dead at 18 months
		23/F	No	Thoracic	GTR	NA	Yes	System	Dead at eight months
		37/F	No	Cervical	GTR	Radiotherapy	Yes	System	Dead at six months
11	Celli et al., 1995 [10]	52/F	No	Thoracic	GTR	No	No	No	Alive and disease-free at 72 months
		68/F	No	Lumbar	GTR	No	No	No	Alive and disease-free at 24 months
		43/M	No	Lumbar	GTR	No	No	No	Alive and disease-free at 72 months
		36/F	No	Thoracic	GTR	Radiotherapy	Yes	No	Alive and disease-free at 48 months
		22/F	Yes	Cervical	GTR	Radiotherapy	Yes	Lung	Dead at six months
		30/M	No	Thoracic	GTR	Radiotherapy	Yes	Lung	Dead at 14 months
17	Acharya et al., 2001 [11]	32/M	No	Cauda Equina	STR	Radiotherapy	No	No	Alive at 18 months
18	Yone et al., 2004 [12]	4/M	No	Cauda Equina	GTR	Radiotherapy, chemotherapy	Yes	Brain	Dead at 21 months
19	Amin et al., 2004 [13]	38/M	No	Cauda equina	Biopsy	Chemotherapy	NA	NA	NA
20	Adamson et al., 2004 [14]	37/M	No	Cervical	STR	Radiotherapy	NA	No	Dead after one year
		30/F	No	Cervical	STR	No	NA	No	Dead after one year
22	Albayrak et al., 2006 [15]	25/M	Yes	Thoracic	GTR	No	Yes	Lung	Alive at seven weeks
23	Chamoun et al., 2009 [16]	5/F	No	Cervical	STR	Radiotherapy, chemotherapy	NA	NA	Alive at four months
24	Qiang et al., 2012 [3]	8/M	No	Cauda equina	GTR	Radiotherapy	Yes	Brain	Dead at 16 months

Patients with primary intradural MPNSTs often present with non-specific symptoms such as localized pain, neurological deficits, and sensory or motor impairments, which can mimic other spinal pathologies, such as schwannomas or neurofibromas [17]. Magnetic resonance imaging is the primary imaging modality and typically reveals a well-circumscribed, heterogeneous mass that shows contrast enhancement, iso-intensity on T1-weighted images, and hyper-intensity on T2-weighted images. However, imaging alone cannot definitively diagnose MPNSTs; immunohistochemical analyses are required for confirmation. Histologically, MPNSTs exhibit spindle-shaped tumor cells with atypical features. Immunohistochemical staining typically shows positive markers such as S-100 and SOX-10, while Ki-67 staining often reveals high proliferation rates indicative of malignancy [18]. Recent research has identified genetic mutations contributing to MPNST pathogenesis, especially alterations in NF1, CDKN2A, and components of the PRC2, including EED, EZH2, and SUZ12. Studies suggest that sequential mutations in these genes drive the transformation from benign nerve sheath tumors to malignant MPNSTs. Loss of function mutations in PRC2 components, which are involved in chromatin modification, are particularly associated with tumor aggressiveness. This genetic profile underscores the potential for targeted therapies in MPNST treatment, with PRC2 mutations being a promising area for therapeutic intervention [18]. Primary intradural MPNSTs pose a treatment challenge due to their aggressive nature, high recurrence rates, and metastatic potential. Gross total resection (GTR) with negative margins is considered the primary therapeutic approach. However, GTR alone often does not prevent recurrence, as the likelihood of local and leptomeningeal spread remains high. Adjuvant radiotherapy, typically with high-dose protocols, has been employed to improve local control and delay recurrence. The use of CIRT has shown promise in MPNSTs due to its superior dose localization and potent cytotoxicity against radioresistant cells. However, clinical evidence for CIRT in spinal MPNSTs remains limited, with only three documented cases​ [5].

Challenges in diagnosis and management

The rarity and aggressive biology of primary intradural MPNSTs make diagnosis and treatment particularly challenging. These tumors can be misdiagnosed as benign intradural masses, such as schwannomas or neurofibromas, due to overlapping imaging characteristics. As such, surgical resection often plays a dual role in both diagnosis and treatment. Complete surgical excision with negative margins is essential for initial management, yet even gross total resection frequently fails to prevent recurrence, which significantly impacts prognosis.

Recurrence and prognosis

Primary intradural MPNSTs of the cauda equina have a high recurrence rate, even with combined surgical and adjuvant therapies. Literature review findings indicate that recurrence can occur as early as weeks to years postoperatively, with cases often demonstrating aggressive local regrowth or distant metastasis. Studies also show that MPNSTs in patients with NF1 may have worse outcomes due to genetic predispositions that favor tumorigenesis [19]. Overall, the prognosis for intradural MPNSTs is poorer than that for other MPNSTs, reflecting the challenges in achieving durable control and the limitations of existing therapies.

Potential for targeted therapies

The identification of genetic mutations in NF1, CDKN2A, and PRC2 components in MPNST pathogenesis opens avenues for targeted therapies. Loss-of-function mutations in PRC2 components (e.g., EED, SUZ12) have been implicated in MPNST development, and therapies targeting EZH2, an enzyme within PRC2, have shown preclinical promise [20]. Future studies should investigate the feasibility and efficacy of EZH2 inhibitors and other targeted therapies in MPNSTs, particularly in cases where traditional treatments offer limited benefits. PRC2-targeted therapy may help mitigate the aggressive nature of these tumors, potentially improving survival rates.

Advances in radiotherapy

Carbon ion radiotherapy has emerged as a promising adjunctive treatment for MPNSTs, especially in cases where conventional radiotherapy may be insufficient. The physical properties of carbon ions allow for precise dose localization, minimizing damage to surrounding healthy tissues and enhancing tumor cell lethality. Limited reports suggest that CIRT, in conjunction with surgical resection, may help in achieving prolonged local control and survival, with some cases demonstrating stability for over a year post treatment. However, evidence for CIRT in spinal and cauda equina MPNSTs is sparse, and larger studies are required to validate its role and efficacy. Given its potential advantages, further research into CIRT’s use in intradural MPNSTs may help define its role as a standard adjunct to surgery [5].

Future research directions

This case, alongside the literature, highlights a critical need for research into both genetic underpinnings and novel therapeutic approaches for primary intradural MPNSTs. Molecular and genetic profiling may allow for the development of individualized therapies targeting specific mutations, such as those in PRC2. Additionally, expanding clinical trials to include therapies like CIRT and targeted molecular agents could provide further insight into the best treatment combinations for these challenging cases. Ultimately, more comprehensive studies are required to improve diagnostic precision, optimize treatment protocols, and extend survival in patients with primary intradural MPNST of the cauda equina.

## Conclusions

This case highlights the rarity and aggressiveness of primary intradural MPNST of the cauda equina. Surgical resection combined with radiotherapy may provide the best chance for local control, but the overall prognosis remains poor. Further research is needed to understand the genetic underpinnings of MPNSTs and to evaluate novel treatment options, including PRC2-targeted therapies and CIRT, to improve outcomes for this challenging condition.
